# Dilated Semantic Segmentation for Breast Ultrasonic Lesion Detection Using Parallel Feature Fusion

**DOI:** 10.3390/diagnostics11071212

**Published:** 2021-07-05

**Authors:** Rizwana Irfan, Abdulwahab Ali Almazroi, Hafiz Tayyab Rauf, Robertas Damaševičius, Emad Abouel Nasr, Abdelatty E. Abdelgawad

**Affiliations:** 1 Department of Information Technology, College of Computing and Information Technology at Khulais, University of Jeddah, Jeddah 21959, Saudi Arabia; 04220818@uj.edu.sa (R.I.); aalmazroi@uj.edu.sa (A.A.A.); 2 Centre for Smart Systems, AI and Cybersecurity, Staffordshire University, Stoke-on-Trent ST4 2DE, UK; 3 Faculty of Applied Mathematics, Silesian University of Technology, 44-100 Gliwice, Poland; robertas.damasevicius@polsl.pl; 4 Industrial Engineering Department, College of Engineering, King Saud University, Riyadh 11421, Saudi Arabia; eabdelghany@ksu.edu.sa (E.A.N.); Aesayed@ksu.edu.sa (A.E.A.)

**Keywords:** CNN, Di-CNN, dilation, DenseNet201, semantic segmentation, parallel feature fusion

## Abstract

Breast cancer is becoming more dangerous by the day. The death rate in developing countries is rapidly increasing. As a result, early detection of breast cancer is critical, leading to a lower death rate. Several researchers have worked on breast cancer segmentation and classification using various imaging modalities. The ultrasonic imaging modality is one of the most cost-effective imaging techniques, with a higher sensitivity for diagnosis. The proposed study segments ultrasonic breast lesion images using a Dilated Semantic Segmentation Network (Di-CNN) combined with a morphological erosion operation. For feature extraction, we used the deep neural network DenseNet201 with transfer learning. We propose a 24-layer CNN that uses transfer learning-based feature extraction to further validate and ensure the enriched features with target intensity. To classify the nodules, the feature vectors obtained from DenseNet201 and the 24-layer CNN were fused using parallel fusion. The proposed methods were evaluated using a 10-fold cross-validation on various vector combinations. The accuracy of CNN-activated feature vectors and DenseNet201-activated feature vectors combined with the Support Vector Machine (SVM) classifier was 90.11 percent and 98.45 percent, respectively. With 98.9 percent accuracy, the fused version of the feature vector with SVM outperformed other algorithms. When compared to recent algorithms, the proposed algorithm achieves a better breast cancer diagnosis rate.

## 1. Introduction

There are numerous cancer types in the world, with breast cancer becoming the leading cause of death in 2020. In 2020, there were 2.2 million new cases of breast cancer reported worldwide. According to the Global Cancer Observatory, 0.68 million people died from breast cancer worldwide, with Asia being the most affected region with a 50.5% ratio [1]. Breast cancer is often not diagnosed until it is advanced because people in middle-income countries have less resources. Prevention strategies, on the other hand, can reduce the risk of death. Indeed, because it has such a negative impact on women’s health, this cancer must be detected in its early stages.

Breast cancer is diagnosed using a variety of imaging modalities. Deep learning and machine learning have been applied in a variety of applications, including renewable energy [2], medical imaging [3], cloud computing [4], agriculture [5,6], fishery [7], cybersecurity [8], and optimization [9]. Medical imaging has seen many advancements in recent years, resulting in non-invasive imaging modalities. Ultrasound, mammography, and magnetic resonance imaging (MRI) are all common medical imaging techniques. Ultrasonic imaging is one of the best techniques because it does not use harmful radiation that can harm the body. It is also sensitive to dense breast masses, which leads to improved detection of cysts from solid tumors, which are usually difficult for the mammography technique to detect [10].

For precise resolution, the frequency range in breast cancer biopsy is between 30 and 60 MHz. The previous consideration for using ultrasonic technology was that it does not result in tissue heating, whereas other techniques, such as X-rays, can cause cancer. Medical professionals prefer a fair amount of information when it comes to exposing patients to ionizing radiation [11].

Furthermore, ultrasonic technology is regarded as a necessary alternative to mammography because it is less expensive, more accurate, more sensitive, less invasive, and takes less time. Sonographers frequently perform manual diagnoses using ultrasonic reports, which is time consuming and can compromise the results if they are inexperienced. Some breast lesions must be identified in these images. To detect these lesions, numerous studies have used segmentation and localization.

Several deep learning algorithms have been used to segment medical images [12]. U-Net is one of the most successful deep-learning-based image segmentation approaches for medical image analysis. It employs the downsampling to upsampling approach for skip connections. The proposed study used this approach in its framework for segmentation purposes, which was inspired by in-depth semantic segmentation. Following lesion segmentation, feature extraction is an essential part of classification because the acquired meaningful features lead to the correct diagnosis of breast cancer [13]. While numerous research papers on feature extraction and classification have been published, radiologists still require reliable, informative potential features for a more powerful cancer diagnosis due to computer experts’ lack of domain knowledge [14].

Artifacts, speckle noise, and lesion shape similarities can be found in ultrasonic images. Breast lesion segmentation remains an unsolved problem as a result of these difficulties [15,16,17]. Existing studies lack vigor, intensity inhomogeneity, artifact removal, and precise lesion segmentation [15,16,18,19]. Because of the deep convolutional process, which extracts rich feature vectors, deep learning-based approaches for semantic segmentation and classification have gained popularity [19,20].

As a result, the aforementioned issues must eventually be considered for automated breast cancer diagnosis in order to improve methods, efficiency, and accuracy [21]. The proposed framework for semantic segmentation of ultrasonic breast images employs dilated factors in convolutional layers.

The main contributions of this study are given below:

- A dilated semantic segmentation network with weighted pixel counts.
- The segmentation method enhanced with the erosion operation.
- Dense features are extracted from the proposed 24-layer CNN, which uses transfer learning to transfer the enriched features to the next layer.
- The fusion of the obtained feature vectors from the segmented image through the Di-CNN.

The rest of the article is structured as follows: Section 2 includes recent related work and the detailed literature review, along with the description of modalities and results. Section 3 presents the materials and methods, including the proposed segmentation approach with the dilated CNN framework and a flowchart of the proposed framework. The experimental results and their analysis are given in Section 4. Finally, the conclusions are made in Section 5.

## 2. Related Work

Several computer-assisted diagnosis systems were developed in previous studies. Some employ segmentation of a specific region of lesions, while others employ direct classification without segmentation. Various segmentation studies have been conducted in the past. A context level set for breast lesion segmentation is proposed. To obtain discriminative information, low-level features are used.

The semantic information is gathered using U-net, and contextual features are then added to create a new energy term [22]. The encoded U-Net approach is used for breast tumor segmentation, and it achieves nearly 90.5 percent dice score on the 510 image dataset. To demonstrate the dominance of attention encodings, the salient attention layers are compared without the salient layers’ approach.

The Hilbert transform was used to reproduce B-mode features from raw images, followed by the marker-controlled watershed transformation to segment the breast cancer lesion [23]. The techniques used, which were solely focused on texture analysis, were very susceptible to speckle noise and other objects.

Following the extraction of shape-based and texture features from the breast lesion, a hybrid feature set was created. Various machine learning classifiers were used to distinguish between cancerous and benign lesions.

The authors proposed a novel second-order subregion pooling network [24] for improving breast lesion segmentation in ultrasound images. Each segmentation network encoder block includes an attention-weighted subregion pooling (ASP) module, which refines features by combining feature maps from the entire image and segmenting from subregions. Furthermore, a directed multi-dimension second-order pooling (GMP) frame was trained in each part of the region to recognize efficient second-order covariance projections by leveraging additional knowledge and multiple feature dimensions.

Similarly, Ref. [25] proposed an effective method for extracting and selecting features. It was suggested that clinical efforts be reduced without segmentation; effective feature selection can improve classification. It proposed using histogram pyramids with a correlation-based features selection approach to extract oriented gradient features. Following that, by combining learned weights with it, the classification was carried out using minimal sequential optimization. The achieved classification sensitivity was 81.64%, with a specificity of 87.76% [25]. A deep learning-based study used privately collected ultrasonic images to feed shape and orientation scores to the quantitative morphological score. Finally, logistic regression-based analysis was carried out, where validation results show good performance in distinguishing ultrasonic breast masses from unnecessary biopsies [26].

Another study used large numbers of data to train deep learning models with B-mode (binary mode) and color Doppler-based dual models. Three databases were selected with the assistance of 20 radiologists for comparison between expert diagnosis and intelligent model-based analysis. The proposed algorithm outperformed all datasets with which it was tested and achieved 0.982 of Area Under the Curve (AUC) [27]. To target multi-view features, an updated Inception-v3 model is proposed that employs coronal views. Following that, the proposed study compared results with HOG and PCA-based methods, which yielded promising results [28].

A second branch temporal sequence network is proposed, which employs two types of data: binary mode and contrast-enhanced ultrasonic image data. Later, this temporal sequence model employs a shuffling method to shuffle sequences rather than to enhance temporal information. The study produced better results than previous studies, according to the findings [29].

Another study employed cutting-edge CNN variants such as InceptionV3, ResNet50, VGG19, and VGG46, with Inception-v3 achieving the most significant results when compared to sonographer interpretation [30]. A morphological and edge-features analysis with a combinational approach is proposed, with a primary focus on the sum of curvatures based on histograms of their shapes. To classify with single morphological features and incorporate edge features, Support Vector Machine (SVM) is used [31]. Some previous works with their dataset details and results are given in Table 1.

Biomarkers-based research is presented in [33] with optimal parameters of tissue elasticity and indirect pathology. Wrapper feature selection with empirical decomposition is also used for feature reduction. Finally, the estimation model takes into account the increasing transformation of carcinogenic tissues. For shape estimation using ultrasonic images, a mathematical model [35] is proposed. The tumor location is combined with an ultrasonic array in this model to extract breast mass information from the image. Tumor size is recognized and calculated in each image, with higher frequency use recommending future work to achieve a higher visual resolution of images. A parallel hybrid CNN approach to classifying into four categories is proposed in [36]. The targeting data remain the same in this method, but the training of the proposed CNN is switched from the same domain data to a different domain data. Data augmentation is used to overcome the overfitting effect. The patch-wise and full-image-wise classifications are reported with 90.5% and 97.4% accuracy.

The authors introduced semantic segmentation [37] with patch merging for segmentation, where the region of interest (ROI) is cropped using diagonal points. After enhancing patches with various filters, superpixels and a bag-of-words model are used to obtain features. To begin, the classification is performed using a neural network, which is then used to improve results. Following that, the k-nearest neighbor (KNN) method is used to improve overall classification performance. The authors propose a U-net-based segmentation in [38] for tissue type classification. The Gauss–Newton Inversion method is then used to reconstruct masses in order to target ultrasonic and dielectric properties. When compared to previously proposed tissue type classification studies, the proposed algorithm outperforms them all.

A reconstruction method based upon the natural frequency of ultrasonic images is proposed in [34]. Raw data are used to create small patches and amplitude samples. For the classification of relevant instances, radio frequency data amplitude parameters known as Nakagami are used. Using contrast-enhanced perfusion and patterns, the author proposed breast cancer molecular subtypes [39]. The proposed improvement is important in the differential diagnosis of breast cancer subtypes. Statistical analysis is used to evaluate targeted subtypes using decision-making variables such as means and standard deviation. For the analysis of breast ultrasonic image features, a privately collected dataset of patients is used. The features are extracted using sonographers.

A multivariate analysis is performed in [40] to diagnose breast cancer to get the confidence interval of 95% of the area under the curve (AUC), which is shown for the primary cohort as 0.75 and 0.91 for the external cohort. This study achieved 88% sensitivity. Furthermore, the authors used real-time burn ultrasonic image classification using the texture GLCM features [41]. Different temperature level is used for pair-wise classification of binary mode slices.

Another study uses Faster-RCNN for lesion localization [19], where in the absence of available datasets, transfer learning-based features are used. To evaluate the segmented lesions, detection points and intersection over union (IoU) are used. The RGB approach improves dataset recall more than the other approaches. An extensive texture analysis discriminant, and without discriminate analysis features of GLCM, AGLOH for comparative analysis of breast cancer classification is presented in [42].

The authors carried out ensemble learning using ResNet, VGGNet, and DenseNet [43], achieving 94.62% accuracy with 92.31% sensitivity. Another study uses a similarity checking network named Siamese CNN. Similarly, the chemotherapy-based general features are used from ultrasonic images with logistic regularization [44]. The AUC is used to evaluate the proposed method, which achieved 0.79 without prior and 0.84 with prior. The study showed an improvement compared to the morphological features.

For breast cancer classification based on histology images, the authors developed a method based on deep CNNs [32]. The dataset used for the task was the ICIAR 2018 dataset, which has Breast Cancer Histology Images with a hematoxylin and eosin-stained breast histology. Several deep neural network architectures and a gradient boosted trees classifiers were used in their approach. They recorded an accuracy of 87.2% for the four-class classification task. At the high-sensitivity operating stage, they recorded 93.8%, 97.3% AUC, 96.5% sensitivity, and 88.0% specificity for a two-class classification task to detect carcinomas.

## 3. Methodology

**Figure 1:**
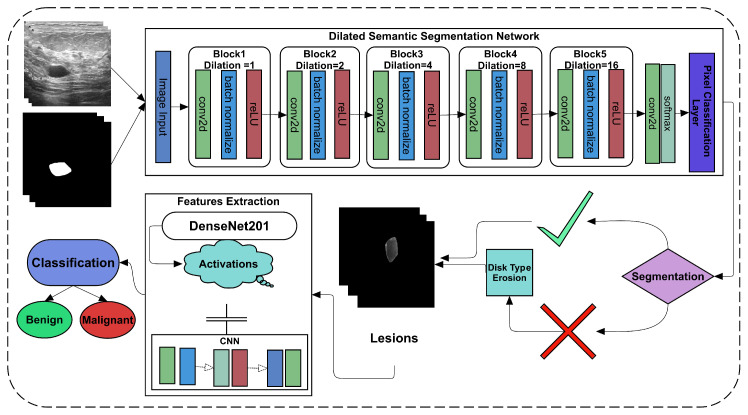
Flowchart of proposed framework.

In the recent era, many studies have suggested employing deep learning as it performs well in medical imaging diagnosis and other image recognition challenges [10,20]. A new dataset of ultrasonic imaging with given label masks is used to solve segmentation and classification challenges [45]. The study used dilated factors in convolutional layers to convolve more appropriately than the simple operation of convolution. The proposed Dilated Convolutional Neural Network (Di-CNN) accurately detects ultrasonic breast tumors. However, the results obtained using simple CNN were not promising to get the same tumor. Therefore, we employed the erosion operation with disk type structuring element on the image with an optional area size operation to get the same tumor. A flow chart of the proposed framework is given in Figure 1.

With a proposed 24-layer CNN, the final segmented tumors are used to extract features. To obtain the transfer learning-based features, another pre-trained ImageNet variant, called DenseNet201, is used. To classify the malignant and benign nodules, both feature extraction approaches are used with and without fusion.

### 3.1. Segmentation

The ROI is extracted from an input image during segmentation. Pixel-level classification, also known as semantic segmentation, is used to extract tumors from given ultrasonic images. Feature vectors are extracted from pixel intensity levels in semantic segmentation. To achieve the goal of tumor extraction, the proposed Di-CNN is further supported by morphological operations.

#### 3.1.1. Dilated Semantic Convolutional Neural Network

**Figure 2:**
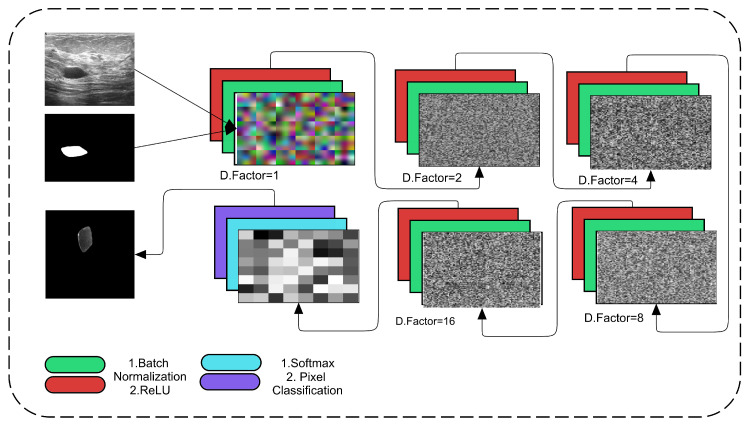
Proposed Dilated Semantic CNN architecture.

The proposed framework employs the regular convolutional operation with the dilation factor as a support. This dilation is increased in each subsequent convolutional layer to obtain more accurate spatial information about tumors and their surroundings. Figure 2 depicts the proposed dilated semantic CNN architecture.

In the proposed Di-CNN, we used five main convolutional blocks, each of which consists of one convolutional layer with batch normalization and a ReLU activation layer. Dilated factors are enriched in these convolutional layers. In even numbers, the dilation factor is increased up to 16. The convolutional kernel’s filter size is 3 × 3 with padding of the same for all orientations. The total number of filters that are used in the layer is 64. The convolutional operation is calculated as usual by convolving each 3 × 3 patch with an increase in the number of rows per column until the digital lattice finishes its end pixel. These convolutional operations are aided by dilation, which employs spacing to cover all spatial information. Dilation factor 1 denotes that the 3 × 3 size factor is added centrally in the first convolutional block.

**Figure 3:**
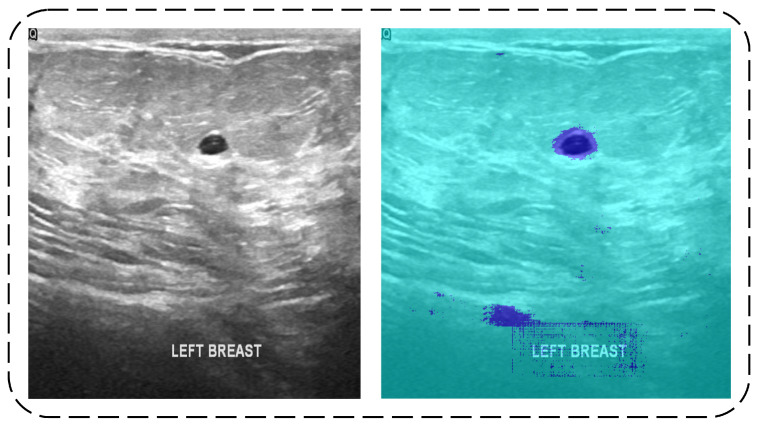
Original Image (**left**) and Di-CNN Prediction (**right**).

Progress in factor from 1 to 2 gains space in the 3 × 3 kernel to the image corner’s extent centrally. However, this spacing is increased up to 16 with the support of batch normalization and ReLU activations. The proposed CNN applies 500 epochs for training the model. It takes a considerable time while training. The dilation factor is proposed by [46], and it uses the following Equation (1). The dilated factor is given as (l)×(k). The *l* represents the number or value of dilation as a factor assigned, where *f* is a discrete function with filter size. However, the results of the proposed Di-CNN are shown in Figure 3.

(1) $$(f\times \left(\left(l\right)\left(k\right)\right)\left(P\right)=\sum_{s+lt=p}f\left(s\right)k\left(t\right)$$

The input image has two areas with two colors, as shown in Figure 3. The predicted tumor class pixels are represented by the dark blue color. However, some images in the testing data contain some noise in the predicted results, which were later covered by the erosion operation.

#### 3.1.2. Erosion

**Figure 4:**
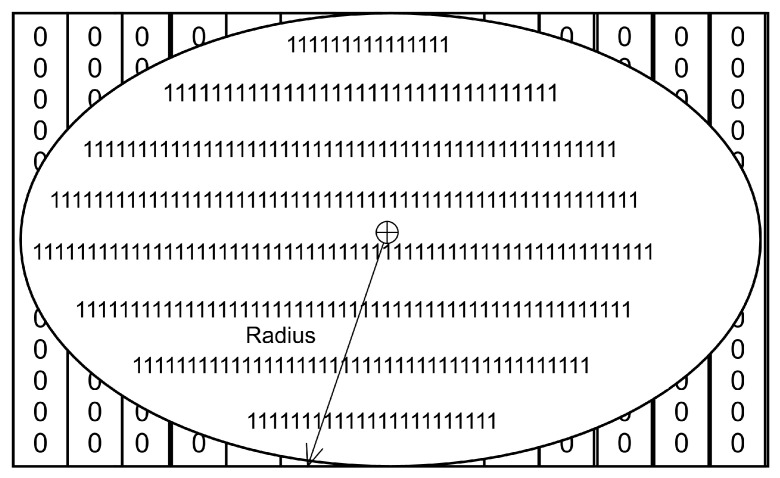
Disk structuring element.

In image processing, erosion is a fundamental operation. It is used to target some specific morphology in image structuring. In our case, the tumor resembles the shape of a disk in most images, which resulted in promising results in predicting the same tumor. The disk-type erosion is used to obtain a radius value in order to centrally map a disk-like object on a given digital binary image. It removes all other noise from the image rather than only that of disk shape. The erosion equation is shown in Equation (2) [47]. The structure of the disk is represented in Figure 4.

(2) $$I⊖E_{str}=\left\{z\right|\;\left({E_{str})}_{z}\;\subseteq I\right\}$$

*I* in Equation (2), the input image has a lesion in it where the $E_{str}$ is the structuring element that has the disk-like structure. The erosion of *I* by $E_{str}$ shows the lesion containing in it where z is the set of points.

**Figure 5:**
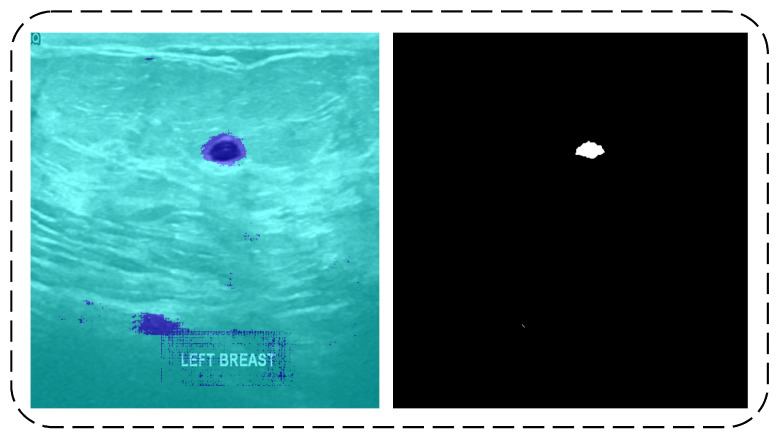
Di-CNN predicted results (**left**) and result after erosion operation (**Right**).

The shallow image of the disk-type element is shown in the input image, which uses a radius to specify the disk size to map on the image. The background image is a binary image in which white pixels with one value form a disk-like structure. To remove noise, this structure is later mapped to predicted tumor pixels. Figure 5 depicts the results of the erosion operation.

**Figure 6:**
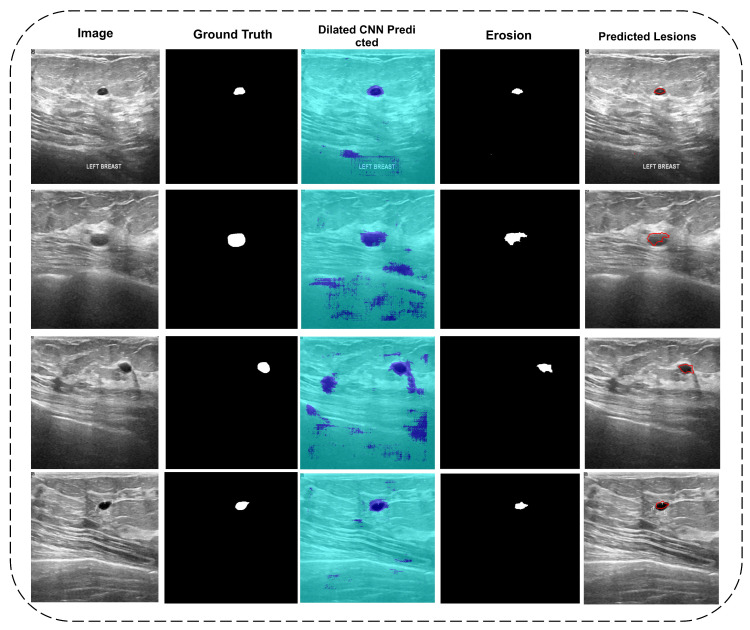
Column 1: Input images; Column 2: Actual mask; Column 3: Di-CNN predicted; Column 4: Erosion operation; Column 5: Real vs. predicted mapping.

The erosion operation begins at the top left side of the given binary image and maps the structuring element to the right by increasing row seeding. A similar shape with some intensity holes is required to eliminate the white pixels. Image noise is removed in this manner. Tumors of the disk type are predicted shaped or actual ground truth labeled. The image was not clear throughout due to the use of erosion to remove such significant areas. The proposed framework may use the area size operation and some of the images if desired. The final tumor segmentation is outlined in the red to map on authentic images to show actual and predicted lesions on images. Figure 6 shows some of the image results from input to final tumor.

Figure 6 representing five columns. The first column shows the original images, while the second columns contain ground truth labels images of the dataset given. The third column shows the results predicted by Di-CNN where some noises were also predicted, as shown in Figure 4. To remove the noise, an erosion operation with a radius range of 5–20 is performed to cover various types of tumors from both classes. The morphology of the exact tumor may be disturbing, but it is preferable to image noise. The fourth column depicts the final eroded mask, which was later mapped onto the original image to obtain the tumor portion. The fifth column shows the final outline of eroded lesions in original images.

The final eroded masks were used to process the feature extraction as segmented lesions. Each aspect of the features, such as shape, localization, and intensity type, was covered by the in-depth learning features.

### 3.2. Features Extraction

The features extraction phase is an important step in obtaining critical information about the malignant and benign classes. Both classes, however, have distinguishing features. Many previous works on feature extraction have been developed, but they may not cover every aspect of geometry, intensity, and localization. Deep learning is used in the proposed study for this purpose. Two types of approaches are used to extract these features.

#### 3.2.1. DenseNet201 Transfer Learning Features

A pre-trained network with 201 layers superimposed 1000 different types of objects. It can be transferred from one dataset to another of the same or lesser size and used to classify objects. The 201-layer deep-learning model, on the other hand, can activate some other image datasets using deep, dense layer models.

Similarly, the proposed study makes use of transfer learning on the DenseNet201 network. The fully connected layer fc1000 is used to extract features from an ultrasonic image dataset. Because there were 647 images, both malignant and benign, the final vector for both images is 647 × 1000. These row-wise features are used to create a final matrix for classification.

#### 3.2.2. Convolutional Neural Network

**Figure 7:**
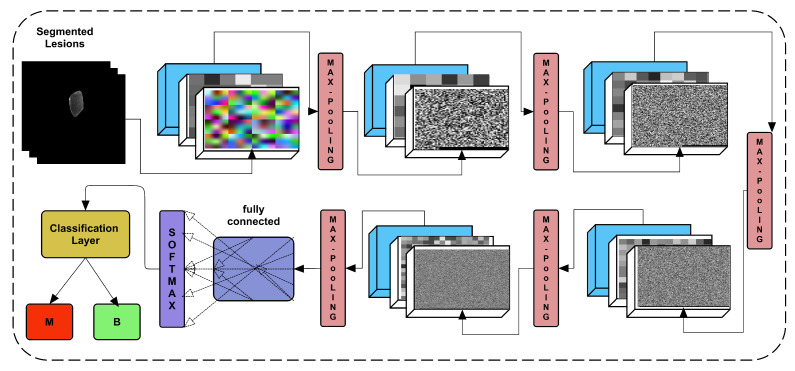
Proposed CNN architecture for classification.

The proposed study offered a 24-layer architecture trained on training images of both classes and later on testing data to use more in-depth learning of lesions images. Figure 7 depicts the architecture of the proposed framework, including the weights of the layers.

Convolution, batch normalization, and ReLU are among the five main blocks in the proposed CNN. Each block output is subjected to additional max pooling. The convolutional kernel is defined as 3 × 3, with the number of filters in each block increasing from block-1 to block-5. The block-1 convolutional layer has 16 filters with the same padding on all four sides (left, right, top, and bottom). This number of filters is higher than N+N. If several filters are used as *N*, then the number of filters is multiplied by the number of blocks to get the following number of filters for blocks 2, 3, 4, and 5. Therefore, the number of filters used are 16, 32, 64, 128, and 256. However, the five max pooling operations are added at the end of each block. The max-pooling operation remains the same as the 2 × 2 kernel with stride=2.

#### 3.2.3. Feature Fusion

Both extracted feature vectors are then fused into a single feature vector using parallel concatenation. The 647 × 1000 feature vector f1 of dense features and second feature vector f2 647 × 2 of proposed CNN “fc” layer activated vector are concatenated in parallel. Both feature vectors then finally make the final 647 × 1002 f3 vector.

### 3.3. Classification

The proposed research utilizes the fused feature vector and fed into multiple machine-learning classifiers to classify the healthy and unhealthy images. The f1 vector classification employing 10-fold cross-validation, 70%-30%, and 50%-50% splits is performed. Further, the f2 vector is fed to the same classifiers for classification, and then lastly, both vectors undergo fusion using the f3 vector. Moreover, all approaches are validated using three cross-validation approaches.

## 4. Results and Discussion

The proposed study made use of a dataset that was divided into three categories: benign, normal, and malignant. Because there was no mask label in the standard images, two malignant and benign images were used. In the tumor segmentation, a total of 647 images from both classes were used, and 133 normal instances were not included during the segmentation. All images and masks used were not the same in size. Therefore, data augmentation was utilized to make equal sizes of all images as 512 × 512. The employed dataset is publicly available at [45]. The dataset description is given in Table 2.

### 4.1. Di-CNN Evaluation

Use of a 19-layer dilated enriched CNN is proposed for semantic segmentation. Three different types of networks were trained, and the best one was chosen because it outperformed the other two. In segmentation, the evaluation measures Intersection over Union (IoU), global accuracy, F1-score, and mean accuracy were used. Mean-IoU is the most relatable measure of a segmentation network; it is calculated by taking the intersection of binary classes in both masks and using ground truth labels on predicted labels pixel count. The Mean-IoU and Global Accuracy, on the other hand, produced good results, as shown in Table 3.

The accuracy obtained using the proposed Di-CNN was 80.20% for two classes as background and tumor classes. The IoU of all images was scored as 52.89%, where the weighted IoU was used by giving weights to both classes, which allowed us to get more accurate results. The training parameters are further explained in Table 4. The calculation for IoU, accuracy, and BF-score measures are given in Equations (3)–(5) [48].

(3) $$Intersection\;Over\;Union\;\left(IoU\right)=\;\frac{predicted\;mask\;⋂\;actual\;mask}{predicted\;mask\;⋃\;actual\;mask}\;$$

(4) $$Accuracy=\;\frac{\sum_{i=1}^{L}\%\;of\;{Pred}_{L}}{\sum_{i=1}^{L}\%\;of\;{Actual}_{L}}$$

(5) $$BF\;Score=\frac{2\times [\left(Precision\right)\left(Recall\right)]}{Precision\cup Recall}$$

All training parameters of the proposed Di-CNN (see Table 4) show a higher number of epochs and batch sizes, confirming the utility of Di-CNN. For the hyperparameter optimization, we used gradient descent with momentum as an optimizer. However, we used a variant of the proposed CNN to extract features from its fully connected layer.

### 4.2. DenseNet201 Activations Based Classification

Activations on a fully connected layer yielded a feature vector based on an augmented DenseNet201 image input layer dataset. Table 5 displays the results of various support vector machine variants classifiers.

It can be seen that the effects of different pre-trained CNN classifiers using f1 are significantly improved in classifying real positives in given datasets. This significant performance, however, is based on a single data split chunk. We used the K-fold cross-validation to assess the validity of the proposed framework. The classifiers are validated using 10-fold cross-validation as shown in Table 6.

The results of 10-fold cross-validation are the same as in the case with fewer testing data (refer to Table 5). However, to check other feature results, the single CNN activated features are tried first using the f2 feature vector.

### 4.3. CNN Activations based Classification

The single f2 feature vector was used on the same SVM variants using 10-fold and 70-splits to cross-check the proposed approach’s performance. The results of the 70%-30% splits ratio are shown in Table 7. The proposed CNN activated feature layer-based features were used, which showed less accurate results than the results given in Table 5 and Table 6.

Cubic–SVM shows promising results in all approaches with better accuracy, sensitivity, Precision, and F1-Score. The 10-fold validation using the f2 vector is shown in Table 8.

Table 6 also shows the lower performance of f2 feature vector-based classification in all evaluation measures. The study used both f1 and f2 feature vectors to fuse in parallel to make a new feature vector. The reason for including both feature vectors is because the most valuable local features of dataset-specific are used in the proposed CNN as it trained for 500 epochs.

### 4.4. Parallel Fusion

The parallel concatenation of the f1 and f2 feature vectors resulted in a new f3 feature vector of size 647 × 1002. The f3 vector is further fed to all previously used SVM variants. The 70%-30% split-based and 10-fold cross-validation-based classifications are shown in Table 9 and Table 10.

**Figure 8:**
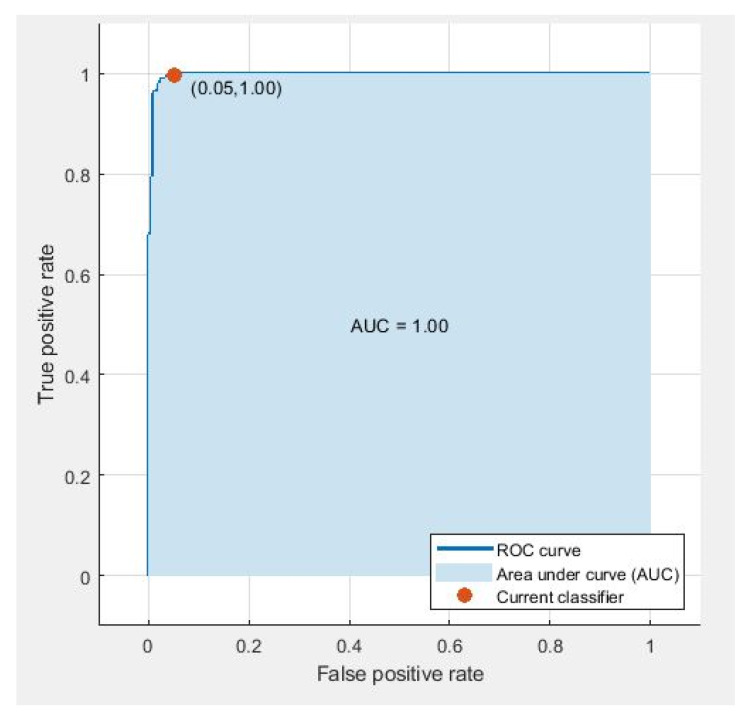
ROC curve using parallel fusion using 10-fold classification o LSVM.

CNN and DenseNet-based fused feature vectors show similar sensitivity, indicating that both networks have similar performance with a similar number of per-class instances (see Table 9 and Table 10). It shows that the proposed feature vectors are robust enough to identify benign and malignant tumors. However, the results in terms of AUC remain 1.00 for all cases in 10-fold cross-validation, as shown in Figure 8. The evaluation measures used for the classification were calculated using Equations (5)–(9).

(6) $$Accuracy=\frac{\left[TP\cup TN\right]}{\left[TP\cup FP\cup TN\cup FN\right]}$$

(7) $$Sensitivity=\frac{TP}{\left[TP\cup FN\right]}$$

(8) $$Specificity=\frac{TN}{\left[TN\cup FP\right]}$$

(9) $$Precision=\frac{TP}{\left[TP\cup FP\right]}$$

**Figure 9:**
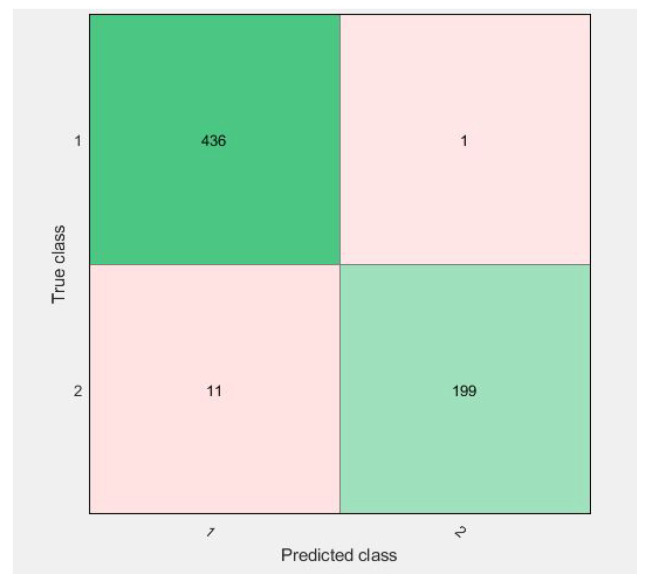
Confusion matrix of parallel fusion using 10-fold classification with LSVM.

**Figure 10:**
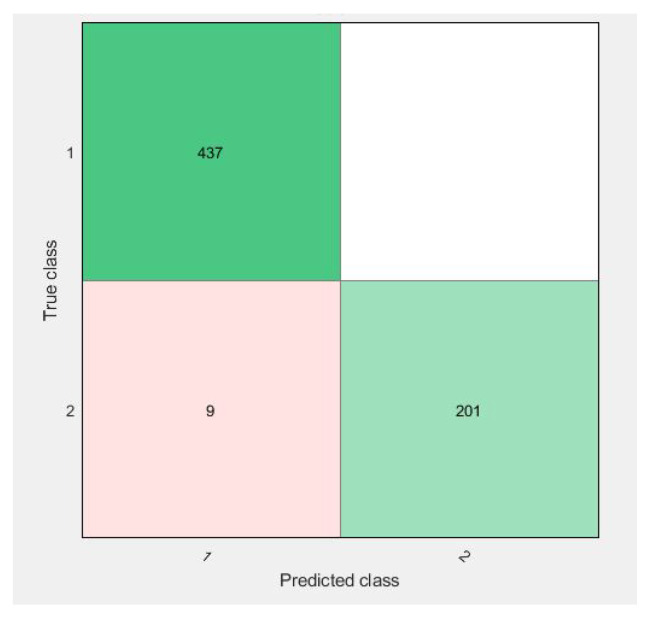
Confusion matrix of parallel fusion using 10-fold classification with QSVM.

**Figure 11:**
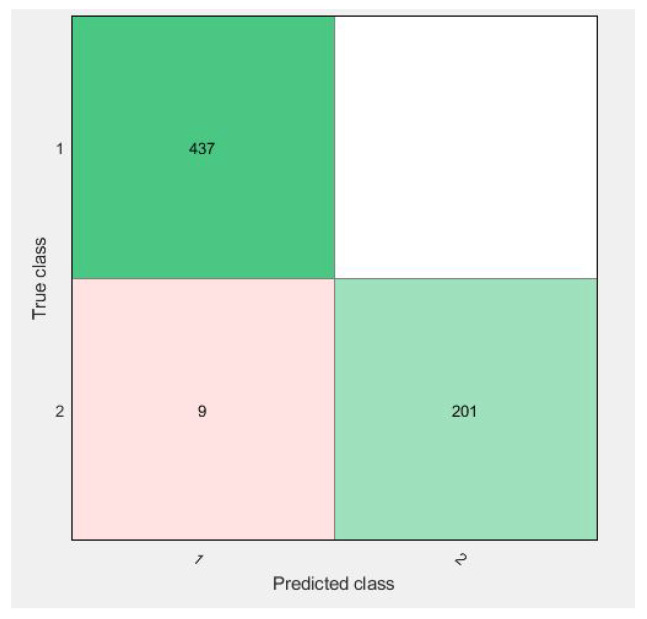
Confusion matrix of parallel fusion using 10-fold classification with Cubic–SVM.

**Figure 12:**
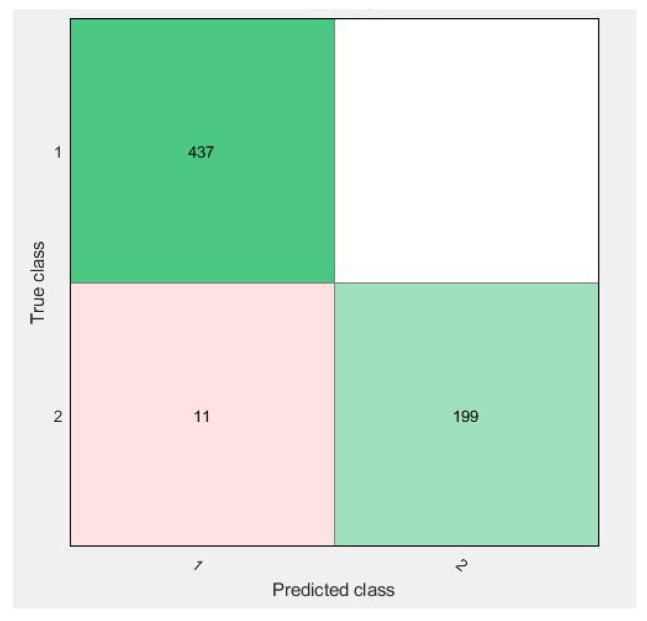
Confusion matrix of parallel fusion using 10-fold classification with SVM–Medium-Gaussian.

Confusion matrices for 10-fold cross-validation using the f3 vector are visualized in Figure 9, Figure 10, Figure 11 and Figure 12.

All of the figures show excellent results for the correct diagnosis of benign cases, whereas malignant cases may require more information for classification using SVM variants. There were 647 total cases in diagonal, with 636 cases correctly diagnosed and 11 cases of a malignant class misclassified. However, when compared to previous studies, the proposed study achieves dominant results.

### 4.5. Discussion

**Figure 13:**
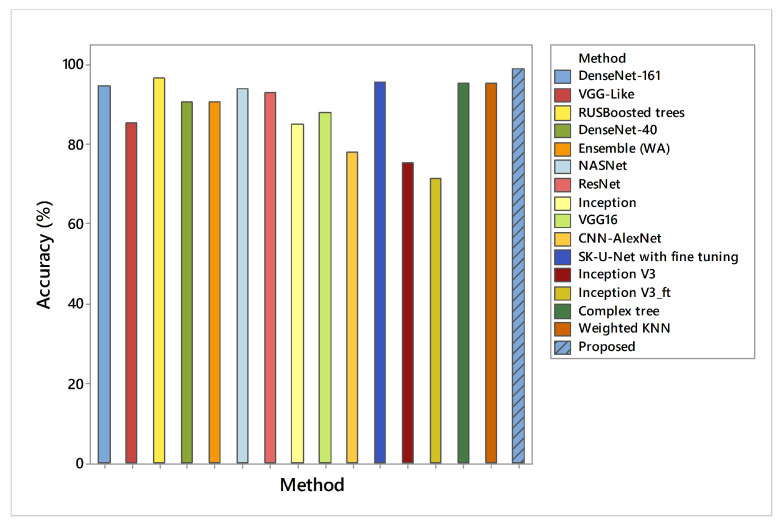
Accuracy comparison of Di-CNN with recent state-of-the-art algorithms on the same dataset.

The results presented in the preceding sections had a significant impact on the use of the proposed methodology for breast cancer detection. The proposed work has generated a lot of confidence due to its validation with 10-fold cross-validation. In addition, each feature vector is evaluated separately on different SVM variants. In classification using the first feature vector, the maximum accuracy (98.97%) was achieved by SVM–Medium-Gaussian with a 70%-30% split ratio to verify that the same vector was tested on 10-fold validation, which reduces the overall accuracy measure by a 0.25–0.5 range. However, to achieve better confidence about used dataset features only, a 24-layer CNN architecture was proposed with the same parameters mentioned in Table 3. However, CNN as a classifier reached up to 80% results only on validation data, which is not discussed where the activations on all data using its ‘fc’ layer were used, which shows higher results than when using the simple CNN features. This feature vector was tested on the same SVM variants with 70%-30% and 10-fold cross-validation. A comparison of Di-CNN with recent state-of-the-art algorithms on the same dataset is given in Figure 13.

We can observe that (see Table 8) feature vectors extracted from CNN-activated layers exhibit intermediate recall rates on 10-fold cross-validation for almost all classifiers except SVM–Medium-Gaussian with 97.25% recall. This phenomenon indicates that SVM–Medium-Gaussian returns more relevant instances during validation than other classifiers. However, cubic SVM appeared as the worst performer in this part of the experiment, with 81.61% accuracy.

The maximum accuracy was 89.18%, with a 96.95% sensitivity value. However, the same feature vector was used for 10-fold cross-validation, which achieves 90.11% accuracy with a 97.25% sensitivity value. Moreover, to obtain both types of feature effects in order to become more confident in our results, the proposed study concatenates both feature vectors, which later pass on to the same classifiers. The same 70–30 splits were applied and achieved 98.97% accuracy with a 100% sensitivity value. The 10-fold validation achieved better results with a maximum accuracy of 98.76% with the same 100% sensitivity.

Similarly, we can see (see Table 6) that feature vectors extracted from DenseNet201 network using transfer learning display low precision (94.76%) with LSVM compared to the other classifiers. QSVM and cubic SVM outperformed other classifiers with a 95.71% precision rate each. All experimental results presented in Table 6 indicate that more of the classifiers detected relevant instances as a positive class than the negative target class.

Our results are compared with some current state-of-the art studies on breast cancer identification, demonstrating that the proposed research has better results than existing algorithms, as shown in Table 11. One study [14] worked on fuzzy interpolative reasoning and selected features using a feature-ranking technique, but it achieved 91.65% accuracy with less sensitivity. Similarly, a deep learning-based ultrasonic image classification, proposed by [22], used submodules with parameter selection to achieve a 96.41% accuracy. Another research study [31] used SVM, KNN, Discriminant Analysis, and random forest classifiers and achieved 82.69%, 63.49%, 78.85%, and 65.83% accuracy, respectively. The last research [43] in Table 9 used ensemble learning with CNNs such as DenseNet-X and VGG to identify breast cancer. It achieved 90.77% accuracy on ultrasonic breast images.

Feature vector fused from activated CNN and DenseNet201 (see Table 10) combined with SVM–Medium Gaussian shows the highest recall rate of 100% on 10 fold cross-validation. All classifiers obtained the same recall rate except the cubic SVM with 99.55%.

As in Figure 9, the confusion matrix has 12 cases in total that wrongly predicted using Linear-SVM, wherein Cubic and Quadratic SVMs have a total of nine results of malignant class being predicted incorrectly. Figure 12 have shown 11 wrongly predicted results of the malignant class in the benign class using the QSVM classifier. Figure 10 represents nine wrongly predicted instances when the feature vectors were fed to SVM–Medium-Gaussian. The difference between misclassification results in Figure 9 and Figure 11 is that, while both have the same number of misclassifications, in Figure 9, the wrongly predicted cases belong to both classes, while Figure 11 shows only one wrong class result, which can lead us to say that the prediction of correct positives increases in the proposed methods. From Figure 13, the RUSBoosted trees appeared as the second-best algorithm for ultrasonic breast image classification after Di-CNN with 96.60% accuracy.

The proposed framework’s limitation could be its failure to use the feature selection technique. Both activated CNN feature vectors and transfer learning-based CNN feature vectors should be fed into the feature selection approach to eliminate the irrelevant features, resulting in a vector with only the most relevant feature, which can improve performance. However, we intend to cover feature selection techniques in future work.

## 5. Conclusions

Breast cancer is becoming increasingly lethal. In developing countries, the death rate is rapidly increasing. As a result, early detection of breast cancer is critical, resulting in a lower mortality rate. We used pixel-level semantic segmentation of ultrasonic breast lesions with dilated factors in this study. An ultrasonic imaging dataset based on masks was used. Following the segmentation phase, the extracted lesions were subjected to an erosion and size filter to remove noise from the segmented lesions when compared to ground truth masks. Finally, for transfer learning features, the DenseNet201 deep network was used, and for feature activations, a proposed CNN was used. Both individual and fusion-based feature vectors were validated using the SVM classifier variants on two validation techniques. However, the final comparison showed a greater improvement in terms of correctly identifying true positives. The accuracy of CNN activated feature vectors and DenseNet201 activated feature vectors combined with the SVM classifier were evaluated, achieving 90.11% and 98.45%, respectively. With 98.9% precision, the fused version of the feature vector with SVM outperformed other algorithms.

In the future, we intend to use more data to work on similar breast cancer identification. The proposed framework of CNN for semantic segmentation and classification may also be improved with the hyperparameter optimization.

## Figures and Tables

**Table 1:** Detailed summary of related works with their modalities and results.

References	Topic	Modality	Dataset Size	Results
[25]	Classification of malignant tumors in breast ultrasound using an unsupervised machine learning approaches	Ultrasonic	Total = 677 Benign = 312 Malignant = 365	Sensitivity = 81.64% Specificity = 87.76% AUC = 0.847
[26]	Deep learning-based computer-aided diagnosis in screening breast ultrasound to reduce false-positive diagnoses	Ultrasonic	Total = 299 Benign = 256 Malignant = 43	Sensitivity = 89% Specificity = 87%
[28]	Breast cancer classification in automated breast ultrasound using multi-view CNN with transfer learning	Ultrasonic	Total = 316 Benign = 181 Malignant = 135	Sensitivity = 88.6% Specificity = 87.6% AUC = 0.94
[29]	A Temporal Sequence Dual-Branch Network for Classifying Hybrid Ultrasound Data of Breast Cancer	Ultrasonic	Total = 268 Benign = 122 Malignant = 146	Accuracy = 90.2% Recall = 91.4% Precision = 95.2% F1-Score = 93.2%
[31]	Breast tumors recognition based on edge feature extraction using support vector machine	Ultrasonic	Total = 192 Benign = 121 Malignant = 71	Accuracy = 82.69% Sensitivity = 66.67% Specificity = 93.55%
[32]	Deep CNN for Breast Cancer Histology Image Analysis	Histology	Total = 400 Benign = 100 Normal = 100 Situ Carcinoma = 100 invasive carcinoma = 100	Accuracy = 93.08% Sensitivity = 96.07% Specificity = 88.00%
[33]	RATE-iPATH: On the design of integrated ultrasonic biomarkers for breast cancer detection	Ultrasonic	Total = 139 Fibroadenoma = 71 Malignant = 44 Inflammation = 24	Accuracy = 99.28% Sensitivity = 100% Specificity = 98.95%
[34]	Breast lesion classification based on ultrasonic radio-frequency signals using convolutional neural networks	Ultrasonic	Total = 100 Benign = 48 Malignant = 52	AUC = 0.772
[23]	Identification of Breast Malignancy by Marker-Controlled Watershed Transformation and Hybrid Feature Set for Healthcare	Ultrasonic	Same as used in the proposed study	Accuracy = 96.6% Sensitivity = 94.34% Specificity = 97.7%
[24]	A Second-Order Subregion Pooling Network for Breast Lesion Segmentation in Ultrasound	Ultrasonic	Same as used in the proposed study	Sensitivity = 85.51% Specificity = 87.62%

**Table 2:** Dataset Description.

Categories	Numbers
Dimension	512 × 512
Malignant	210
Benign	437
Normal	133
Total	780

**Table 3:** Di-CNN Testing Results.

Global Accuracy (%)	Mean-IoU (%)	Mean Accuracy (%)	Weighted-IoU (%)	Mean-BF Score
80.20	52.89	79.61	73.83	0.18218

**Table 4:** Di-CNN training parameters.

Parameter	Value
Batch Size	128
Epochs	500
Activation function	Stochastic gradient decent (sgdm)
Training time	Hours:Minutes:Seconds; 197:03:28
Iterations	20,000
Initial Learning Rate	1 × 10${}^{-4}$

**Table 5:** DenseNet201 activated feature based classification using 70%-30% split.

Method	Accuracy (%)	Sensitivity (%)	Specificity (%)	Precision (%)	F1-Score (%)
LSVM	98.45	100	95.24	97.76	98.87
QSVM	98.97	100	96.83	98.50	99.24
Cubic–SVM	98.45	99.24	96.83	96.48	98.86
SVM–Medium Gaussian	98.97	100	96.83	98.50	99.24

**Table 6:** DenseNet201 activated feature based classification using 10-fold cross validation.

Method	Accuracy (%)	Sensitivity (%)	Specificity (%)	Precision (%)	F1-Score (%)
LSVM	98.30	100	94.76	94.76	98.76
QSVM	98.61	100	95.71	97.98	98.98
Cubic–SVM	98.30	99.54	95.71	97.97	98.75
SVM–Medium Gaussian	98.45	100	95.24	97.76	98.87

**Table 7:** CNN activated Feature based classification using 70%-30% split.

Method	Accuracy (%)	Sensitivity (%)	Specificity (%)	Precision (%)	F1-Score (%)
LSVM	89.18	95.42	76.19	89.29	92.25
QSVM	89.18	96.95	73.02	88.19	92.36
Cubic–SVM	89.18	96.18	74.60	88.73	92.31
SVM–Medium Gaussian	88.14	97.71	68.25	86.49	91.76

**Table 8:** CNN activated feature-based classification using 10-fold cross-validation.

Method	Accuracy (%)	Sensitivity (%)	Specificity (%)	Precision (%)	F1-Score (%)
LSVM	89.64	94.28	80.00	90.75	92.48
QSVM	89.34	94.05	79.52	90.53	92.26
Cubic–SVM	81.61	91.53	60.95	82.99	87.05
SVM–Medium Gaussian	90.11	97.25	75.24	89.10	93.00

**Table 9:** DenseNet201 and CNN activated feature fusion based classification using 70%-30% split.

Method	Accuracy (%)	Sensitivity (%)	Specificity (%)	Precision (%)	F1-Score (%)
LSVM	98.45	100	95.24	97.76	98.87
QSVM	98.97	100	96.83	98.50	99.24
Cubic–SVM	98.97	100	96.83	98.50	99.24
SVM–Medium Gaussian	98.97	100	96.83	98.50	99.24

**Table 10:** DenseNet201 and CNN activated feature fusion-based classification using 10-fold cross-validation.

Method	Accuracy (%)	Sensitivity (%)	Specificity (%)	Precision (%)	F1-Score (%)
LSVM	98.45	100	95.24	97.76	98.87
QSVM	98.76	100	96.19	98.20	99.09
Cubic–SVM	98.45	99.54	96.19	98.19	98.86
SVM–Medium Gaussian	98.45	100	95.24	97.76	98.87

**Table 11:** Comparison of previous methods with the proposed method.

Method	Accuracy (%)	Sensitivity (%)	Specificity (%)
DenseNet-161 [43]	94.62	92.31	95.60
VGG-Like [43]	85.38	76.92	89.00
RUSBoosted trees [23]	96.6	94.34	97.70
DenseNet-40 [43]	90.77	88.89	91.49
Ensemble (WA) [43]	90.77	96.67	89.00
DenseNet-121 [43]	88.46	83.78	90.32
NASNet [49]	94	-	-
ResNet [49]	93	-	-
Inception [49]	85	-	-
VGG16 [49]	88	-	-
CNN-AlexNet [49]	78	-	-
SK-U-Net with fine tuning [50]	95.6	-	-
SK-U-Net without fine tuning [50]	94.4	-	-
Inception V3 [51]	75.6	-	-
w/o-GMP [24]	-	81.99	84.36
w/o-subregions [24]	-	83.97	86.35
ChannelRC [24]	-	84.22	86.72
w/o-guidance [24]	-	84.85	87.09
S2P-Net [24]	-	85.51	87.62
VGG-16 [51]	91.9	-	-
Inception V3_ft [51]	71.3	-	-
VGG-16_ft [51]	86.2	-	-
UNet [22]	96.41	95.17	97.34
Complex tree [23]	95.83	92.96	97.24
Weighted KNN [23]	95.36	92.86	96.56
Proposed	98.97	100	96.83

## Data Availability

The dataset used for this study is publicly available at [45].

## References

[1] Sung H., Ferlay J., Siegel R.L., Laversanne M., Soerjomataram I., Jemal A., Bray F. (2021). Global cancer statistics 2020: GLOBOCAN estimates of incidence and mortality worldwide for 36 cancers in 185 countries. CA Cancer J. Clin..

[2] Gao J., Wang H., Shen H. Smartly Handling Renewable Energy Instability in Supporting a Cloud Datacenter. Proceedings of the 2020 IEEE International Parallel and Distributed Processing Symposium (IPDPS).

[3] Albahli S., Rauf H.T., Arif M., Nafis M.T., Algosaibi A. (2021). Identification of Thoracic Diseases by Exploiting Deep Neural Networks. Neural Netw..

[4] Gao J., Wang H., Shen H. (2020). Task failure prediction in cloud data centers using deep learning. IEEE Trans. Serv. Comput..

[5] Oyewola D.O., Dada E.G., Misra S., Damaševičius R. (2021). Detecting cassava mosaic disease using a deep residual convolutional neural network with distinct block processing. PeerJ Comput. Sci..

[6] Rehman Z.U., Khan M.A., Ahmed F., Damaševičius R., Naqvi S.R., Nisar W., Javed K. (2021). Recognizing apple leaf diseases using a novel parallel real-time processing framework based on MASK RCNN and transfer learning: An application for smart agriculture. IET Image Process..

[7] Rauf H.T., Lali M.I.U., Zahoor S., Shah S.Z.H., Rehman A.U., Bukhari S.A.C. (2019). Visual features based automated identification of fish species using deep convolutional neural networks. Comput. Electron. Agric..

[8] Hemalatha J., Roseline S.A., Geetha S., Kadry S., Damaševičius R. (2021). An efficient densenet-based deep learning model for malware detection. Entropy.

[9] Rauf H.T., Malik S., Shoaib U., Irfan M.N., Lali M.I. (2020). Adaptive inertia weight Bat algorithm with Sugeno-Function fuzzy search. Appl. Soft Comput..

[10] Pang T., Wong J.H.D., Ng W.L., Chan C.S. (2020). Deep learning radiomics in breast cancer with different modalities: Overview and future. Expert Syst. Appl..

[11] Latif G., Butt M.O., Al Anezi F.Y., Alghazo J. Ultrasound Image Despeckling and Detection of Breast Cancer Using Deep CNN. Proceedings of the 2020 RIVF International Conference on Computing and Communication Technologies (RIVF).

[12] Ching T., Himmelstein D.S., Beaulieu-Jones B.K., Kalinin A.A., Do B.T., Way G.P., Ferrero E., Agapow P.M., Zietz M., Hoffman M.M. (2018). Opportunities and obstacles for deep learning in biology and medicine. J. R. Soc. Interface.

[13] Li Y., Liu Y., Zhang M., Zhang G., Wang Z., Luo J. (2020). Radiomics with attribute bagging for breast tumor classification using multimodal ultrasound images. J. Ultrasound Med..

[14] Li F., Shang C., Li Y., Shen Q. (2020). Interpretable mammographic mass classification with fuzzy interpolative reasoning. Knowl.-Based Syst..

[15] Amiri M., Brooks R., Behboodi B., Rivaz H. (2020). Two-stage ultrasound image segmentation using U-Net and test time augmentation. Int. J. Comput. Assist. Radiol. Surg..

[16] Vakanski A., Xian M., Freer P.E. (2020). Attention-enriched deep learning model for breast tumor segmentation in ultrasound images. Ultrasound Med. Biol..

[17] Rakhlin A., Tiulpin A., Shvets A.A., Kalinin A.A., Iglovikov V.I., Nikolenko S. Breast Tumor Cellularity Assessment Using Deep Neural Networks. Proceedings of the IEEE/CVF International Conference on Computer Vision Workshops.

[18] Lahoura V., Singh H., Aggarwal A., Sharma B., Mohammed M.A., Damaševičius R., Kadry S., Cengiz K. (2021). Cloud Computing-Based Framework for Breast Cancer Diagnosis Using Extreme Learning Machine. Diagnostics.

[19] Yap M.H., Goyal M., Osman F., Marti R., Denton E., Juette A., Zwiggelaar R. (2020). Breast ultrasound region of interest detection and lesion localisation. Artif. Intell. Med..

[20] Meraj T., Rauf H.T., Zahoor S., Hassan A., Lali M.I., Ali L., Bukhari S.A.C., Shoaib U. (2019). Lung nodules detection using semantic segmentation and classification with optimal features. Neural Comput. Appl..

[21] Pi Y., Chen Y., Deng D., Qi X., Li J., Lv Q., Yi Z. (2020). Automated diagnosis of multi-plane breast ultrasonography images using deep neural networks. Neurocomputing.

[22] Hussain S., Xi X., Ullah I., Wu Y., Ren C., Lianzheng Z., Tian C., Yin Y. (2020). Contextual Level-Set Method for Breast Tumor Segmentation. IEEE Access.

[23] Sadad T., Hussain A., Munir A., Habib M., Ali Khan S., Hussain S., Yang S., Alawairdhi M. (2020). Identification of breast malignancy by marker-controlled watershed transformation and hybrid feature set for healthcare. Appl. Sci..

[24] Zhu L., Chen R., Fu H., Xie C., Wang L., Wan L., Heng P.A. A Second-Order Subregion Pooling Network for Breast Lesion Segmentation in Ultrasound. Proceedings of the International Conference on Medical Image Computing and Computer-Assisted Intervention.

[25] Shia W.C., Lin L.S., Chen D.R. (2021). Classification of malignant tumors in breast ultrasound using unsupervised machine learning approaches. Sci. Rep..

[26] Kim S.Y., Choi Y., Kim E.K., Han B.K., Yoon J.H., Choi J.S., Chang J.M. (2021). Deep learning-based computer-aided diagnosis in screening breast ultrasound to reduce false-positive diagnoses. Sci. Rep..

[27] Qian X., Zhang B., Liu S., Wang Y., Chen X., Liu J., Yang Y., Chen X., Wei Y., Xiao Q. (2020). A combined ultrasonic B-mode and color Doppler system for the classification of breast masses using neural network. Eur. Radiol..

[28] Wang Y., Choi E.J., Choi Y., Zhang H., Jin G.Y., Ko S.B. (2020). Breast cancer classification in automated breast ultrasound using multiview convolutional neural network with transfer learning. Ultrasound Med. Biol..

[29] Yang Z., Gong X., Guo Y., Liu W. (2020). A Temporal Sequence Dual-Branch Network for Classifying Hybrid Ultrasound Data of Breast Cancer. IEEE Access.

[30] Zhang H., Han L., Chen K., Peng Y., Lin J. (2020). Diagnostic efficiency of the breast ultrasound computer-aided prediction model based on convolutional neural network in breast cancer. J. Digit. Imaging.

[31] Liu Y., Ren L., Cao X., Tong Y. (2020). Breast tumors recognition based on edge feature extraction using support vector machine. Biomed. Signal Process. Control.

[32] Rakhlin A., Shvets A., Iglovikov V., Kalinin A.A. Deep Convolutional Neural Networks for Breast Cancer Histology Image Analysis. Proceedings of the International Conference Image Analysis and Recognition.

[33] Hossen Z., Abrar M.A., Ara S.R., Hasan M.K. (2020). RATE-iPATH: On the design of integrated ultrasonic biomarkers for breast cancer detection. Biomed. Signal Process. Control.

[34] Jarosik P., Klimonda Z., Lewandowski M., Byra M. (2020). Breast lesion classification based on ultrasonic radio-frequency signals using convolutional neural networks. Biocybern. Biomed. Eng..

[35] Chang J., Chen Z., Huang Y., Li Y., Zeng X., Lu C. (2020). Flexible ultrasonic array for breast-cancer diagnosis based on a self-shape–estimation algorithm. Ultrasonics.

[36] Alzubaidi L., Al-Shamma O., Fadhel M.A., Farhan L., Zhang J., Duan Y. (2020). Optimizing the performance of breast cancer classification by employing the same domain transfer learning from hybrid deep convolutional neural network model. Electronics.

[37] Huang Q., Huang Y., Luo Y., Yuan F., Li X. (2020). Segmentation of breast ultrasound image with semantic classification of superpixels. Med. Image Anal..

[38] Mojabi P., Khoshdel V., Lovetri J. (2020). Tissue-Type Classification With Uncertainty Quantification of Microwave and Ultrasound Breast Imaging: A Deep Learning Approach. IEEE Access.

[39] Liang X., Li Z., Zhang L., Wang D., Tian J. (2020). Application of Contrast-Enhanced Ultrasound in the Differential Diagnosis of Different Molecular Subtypes of Breast Cancer. Ultrason. Imaging.

[40] Luo Y., Zhao C., Gao Y., Xiao M., Li W., Zhang J., Ma L., Qin J., Jiang Y., Zhu Q. (2020). Predicting Axillary Lymph Node Status With a Nomogram Based on Breast Lesion Ultrasound Features: Performance in N1 Breast Cancer Patients. Front. Oncol..

[41] Lee S., Rahul, Ye H., Chittajallu D., Kruger U., Boyko T., Lukan J.K., Enquobahrie A., Norfleet J., De S. (2020). Real-time burn classification using ultrasound imaging. Sci. Rep..

[42] Kore S.S., Kadam A.B. (2020). A novel incomplete sparse least square optimized regression model for abdominal mass detection in ultrasound images. Evol. Intell..

[43] Moon W.K., Lee Y.W., Ke H.H., Lee S.H., Huang C.S., Chang R.F. (2020). Computer-aided diagnosis of breast ultrasound images using ensemble learning from convolutional neural networks. Comput. Methods Prog. Biomed..

[44] Byra M., Dobruch-Sobczak K., Klimonda Z., Piotrzkowska-Wroblewska H., Litniewski J. (2020). Early prediction of response to neoadjuvant chemotherapy in breast cancer sonography using Siamese convolutional neural networks. IEEE J. Biomed. Health Inform..

[45] Al-Dhabyani W., Gomaa M., Khaled H., Fahmy A. (2020). Dataset of breast ultrasound images. Data Brief.

[46] Yu F., Koltun V. (2015). Multi-scale context aggregation by dilated convolutions. arXiv.

[47] Gonzalez R.C., Woods R.E., Masters B.R. (2008). Digital Image Processing.

[48] Jadon S. A Survey of Loss Functions for Semantic Segmentation. Proceedings of the 2020 IEEE Conference on Computational Intelligence in Bioinformatics and Computational Biology (CIBCB).

[49] Al-Dhabyani W., Gomaa M., Khaled H., Aly F. (2019). Deep learning approaches for data augmentation and classification of breast masses using ultrasound images. Int. J. Adv. Comput. Sci. Appl..

[50] Byra M., Jarosik P., Szubert A., Galperin M., Ojeda-Fournier H., Olson L., O’Boyle M., Comstock C., Andre M. (2020). Breast mass segmentation in ultrasound with selective kernel U-Net convolutional neural network. Biomed. Signal Process. Control.

[51] Lazo J.F., Moccia S., Frontoni E., De Momi E. (2020). Comparison of different CNNs for breast tumor classification from ultrasound images. arXiv.

